# Lipopolysaccharide-binding protein (LBP) can reverse the amyloid state of fibrin seen or induced in Parkinson's disease

**DOI:** 10.1371/journal.pone.0192121

**Published:** 2018-03-01

**Authors:** Etheresia Pretorius, Martin J. Page, Sthembile Mbotwe, Douglas B. Kell

**Affiliations:** 1 Department of Physiological Sciences, Faculty of Science, Stellenbosch University, Stellenbosch, South Africa; 2 Department of Physiology, Faculty of Health Sciences, University of Pretoria, Arcadia, South Africa; 3 School of Chemistry, The University of Manchester, Manchester, Lancs, United Kingdom; 4 The Manchester Institute of Biotechnology, The University of Manchester, Manchester, Lancs, United Kingdom; National Institutes of Health, UNITED STATES

## Abstract

The thrombin-induced polymerisation of fibrinogen to form fibrin is well established as a late stage of blood clotting. It is known that Parkinson’s Disease (PD) is accompanied by dysregulation in blood clotting, but it is less widely known as a coagulopathy. In recent work, we showed that the presence of tiny amounts of bacterial lipopolysaccharide (LPS) in healthy individuals could cause clots to adopt an amyloid form, and this could be observed via scanning electron microscopy (SEM) or via the fluorescence of thioflavin-T. This could be prevented by the prior addition of lipopolysaccharide-binding protein (LBP). We had also observed by SEM this unusual clotting in the blood of patients with Parkinson’s Disease. We hypothesised, and here show, that this too can be prevented by LBP in the context of PD. This adds further evidence implicating inflammatory microbial cell wall products as an accompaniment to the disease, and may be part of its aetiology. This may lead to novel treatment strategies in PD designed to target microbes and their products.

## Introduction

It is widely recognized that that many chronic, inflammatory diseases are accompanied by insoluble amyloid fibril formation [1–7]. Parkinson’s Disease (PD) is one such condition, and is accompanied by amyloid forms of α-synuclein in the substantia nigra pars compacta [8–13]. To this end, systems biology approaches [14–18] have made considerable headway in accounting for the known “Parkinson’s” genes in biochemical terms, with the additional recognition that dysregulated cytokines, oxidative stress and particularly iron dysregulation are also major contributors to disease progression [19–31].

It is rather less widely recognized that PD may also be accompanied by changes in the normal clotting of blood, i.e. it may be a coagulopathy [32–35], albeit the last of these papers suggested that the changes were more in the complement than the coagulation cascade. Indeed, PD in treated patients has been observed to be accompanied by changes in prothrombin time and D-dimer formation (Sato *et al*., 2003), as well as by eryptosis [35]. Focusing on the terminal stages of the coagulation cascade, thrombin removes two fibrinopeptides from fibrinogen, thereby allowing the fibrinogen to self-assemble into insoluble fibrin via a ‘knobs and holes’ mechanism (e.g. [36–40]). There are not otherwise considered to be any major changes in secondary structure of the protein [36, 41–43]. A final crosslinking step catalyzed by Factor XIII, after it too has been activated by thrombin, [44] increases the stability of the clot.

In healthy individuals, the addition of thrombin to platelet poor plasma (PPP) causes the fibres forming the subsequent clot to appear as loosely collected netlike structures (comparable to a plate of noodles or spaghetti) under the scanning electron microscope [45–49]. However, we and others have observed that fibre diameter and morphology change markedly in a variety of vascular and inflammatory diseases, typically producing ‘dense matter deposits’ (e.g. [46, 50–59]).

Even though, as mentioned, there are *usually* no major changes in the secondary structure of the fibre protein during polymerization, it *can* undergo structural changes under certain conditions. Although fibrinogen is not considered to be amyloidogenic, nor is fibrin seen as an amyloid protein, it can nonetheless become so in the presence of a rare mutation in the fibrinogen a chain [60–63]), or by extreme mechanical stretching [64–66]. A very specific feature of amyloid proteins is the formation of a cross-β-sheet structure, perpendicular to the fibres with a characteristic spacing (observable in X-ray reflections) of 4.7–4.8Å (e.g. [4, 7, 67, 68]). In contrast to normal structures, thioflavin T (ThT) binds strongly to them, and fluoresces intensely at 480–520 nm when excited at ~440 nm (e.g. [69–74]). Indeed, following many observations in the SEM of anomalous blood clotting (e.g. [27, 48, 49, 58, 59, 75–77]), we have recently established [77, 78] that this anomalous clotting *is* in fact amyloid in nature.

Previously in our laboratory, we have shown that fibrin amyloid formation occurred in the presence of tiny amounts of bacterial lipopolysaccharide (LPS) (0.2 ng·L^-1^; 1 molecule per 10^8^ fibrinogen molecules), but was abolished when this was added together with a two-fold stoichiometric excess of human LBP (lipopolysaccharide binding protein) [77]. We have particularly emphasised that dormant bacteria are widespread in nature [79–83], and we have argued strongly for a role for dormant bacteria in the aetiology of such diseases [84–91]. Indeed, recent work in mice lends strong support to the view that the gut microbiome can play a major role in the aetiology of PD [11]. Iron is also capable of catalysing anomalous blood clotting [75, 76], and as mentioned previously, there are strong indications for both iron dysregulation [16, 21, 25–27, 92–97] and coagulopathies [58] in Parkinson’s Disease.

The purpose of the present paper was thus to study whether (and to what extent) fibrin-type amyloid in blood varies between suitably matched controls and individuals with Parkinson’s Disease. In addition, we wished to investigate whether LBP affected this in any way, to a potentially therapeutic effect. It became clear that the answers are in the affirmative in both cases.

## Materials and methods

### Ethical statement

Ethical clearance was obtained from the Health Sciences Ethical Committee of the University of Pretoria and written informed consent was obtained from each of the patients, as well as from control donors (ethical number: 80/2013 and reapproved 2015) (available on request). Exclusion criteria for the PD patients were conditions such as asthma, human immunodeficiency virus (HIV) or tuberculosis, and risk factors associated with metabolic syndrome, smoking, and (if female) being on contraceptive or hormone replacement treatment. Exclusion criteria for the healthy population were known inflammatory conditions such as asthma, human immunodeficiency virus (HIV) or tuberculosis, and risk factors associated with metabolic syndrome, smoking, and if female, being on contraceptive or hormone replacement treatment. This population did not take any anti-inflammatory medication. Whole blood of all participants was obtained in citrate tubes and platelet poor plasma (PPP) was used for confocal and SEM experiments. The methods were carried out in accordance with the approved guidelines. Blood was collected and methods were carried out in accordance with the relevant guidelines of the ethics committee. We adhered strictly to the Declaration of Helsinki.

### Sample population

In this study, 19 healthy, age-controlled individuals, of whom 9 were spouses of some of the Parkinson’s Disease (PD) individuals, and 26 individuals diagnosed with PD, were included. The PD patients were diagnosed by a Neurologist and the Unified Parkinson’s Disease Rating Scale (UPDRS) was used in this diagnosis. On the day of blood collection, the Hoehn and Yahr scale was used by a clinician to rate the relative level of the PD disability. Margaret M. Hoehn and Melvin D. Yahr developed the Hoehn and Yahr scale to scale practically the severity of PD at the time of treatment, and thereby determine whether the medication or treatment that is used influences the rate of the progression of the disease [98]. Many studies thereafter have used this method in scaling the severity of movement disorders [99–103].

### LPS-binding protein

A final LPS-binding protein (LBP) exposure concentration of 2 ng·L^-1^ LBP was used [77]. LBP was purchased from Sigma (recombinant product SRP6033; >95% pure). Previously we reported that LBP added to healthy plasma does not change or affect the structure of healthy fibrin viewed with scanning electron microscopy (SEM) [104]. We also previously showed with confocal microscopy that healthy PPP with added LBP (after addition of thrombin) showed little change in fluorescent (amyloid) signal [77].

### Airyscan and scanning electron microscopy

PPP was prepared from whole blood collected in citrated tubes, from both healthy and PD individuals. For Airyscan preparation, we added Thioflavin T (ThT) at a final concentration of 5 μM to 200 μL of various prepared PPP samples and incubated it (protected from light) for one minute. This step was followed with the addition of thrombin, added in the ratio 1:2 to create extensive fibrin networks. A coverslip was placed over the prepared clot, and viewed immediately using a Zeiss LSM 510 META confocal microscope with a Plan-Apochromat 63× and 100×/1.4 Oil DIC objective and super-resolution (Airyscan) capabilities. The Airyscan detector increases the resolution by a factor of 1.7, achieving super-resolution of 140 nm. Excitation was at 488 nm and emitted light was measured at 505–550 nm. In addition, PPP from PD individuals was incubated with LBP (final concentration 2 ng·L^-1^) for 10 minutes, followed by ThT and clot preparation as for the healthy and naïve PD PPP. Clots were also prepared for SEM analysis, but after addition of thrombin, clots were washed, fixed in 4% formaldehyde and prepared for SEM according to known SEM preparation methods (e.g. Pretorius *et al*. 2013b). Samples were viewed using a Zeiss cross beam electron microscope to study fibrin fibres.

### Statistical analysis and data sharing

We used a One-Way ANOVA with Holm-Šídák Multiple Comparison’s Test for the SEM analysis (controls vs. PD vs. PD with added LBP), comparing the mean of each column with the mean of every other column (GraphPad 7). The paired t-test analysis was done for the Airyscan analysis (PD with and without LBP). For each picture, we obtained the histogram of intensities (8-bit scale) using the *histogram* function of ImageJ. From this, we calculated the coefficient of variation (CV, as standard deviation/mean) (see [104] for detailed methodology). The experiments were done blinded and data capturing and analysis of micrographs were performed by different co-authors.

All relevant data (Excel data files, lab SOPs, and the raw data graphpad stats file) to replicate the study's findings are available from figshare: https://doi.org/10.6084/m9.figshare.5844738. Full confocal and electron microscopy micrographs files are available from the corresponding author or at the following link: https://1drv.ms/f/s!AgoCOmY3bkKHmWY8VijKiQ-8_5RY."

## Results

**Fig 1:**
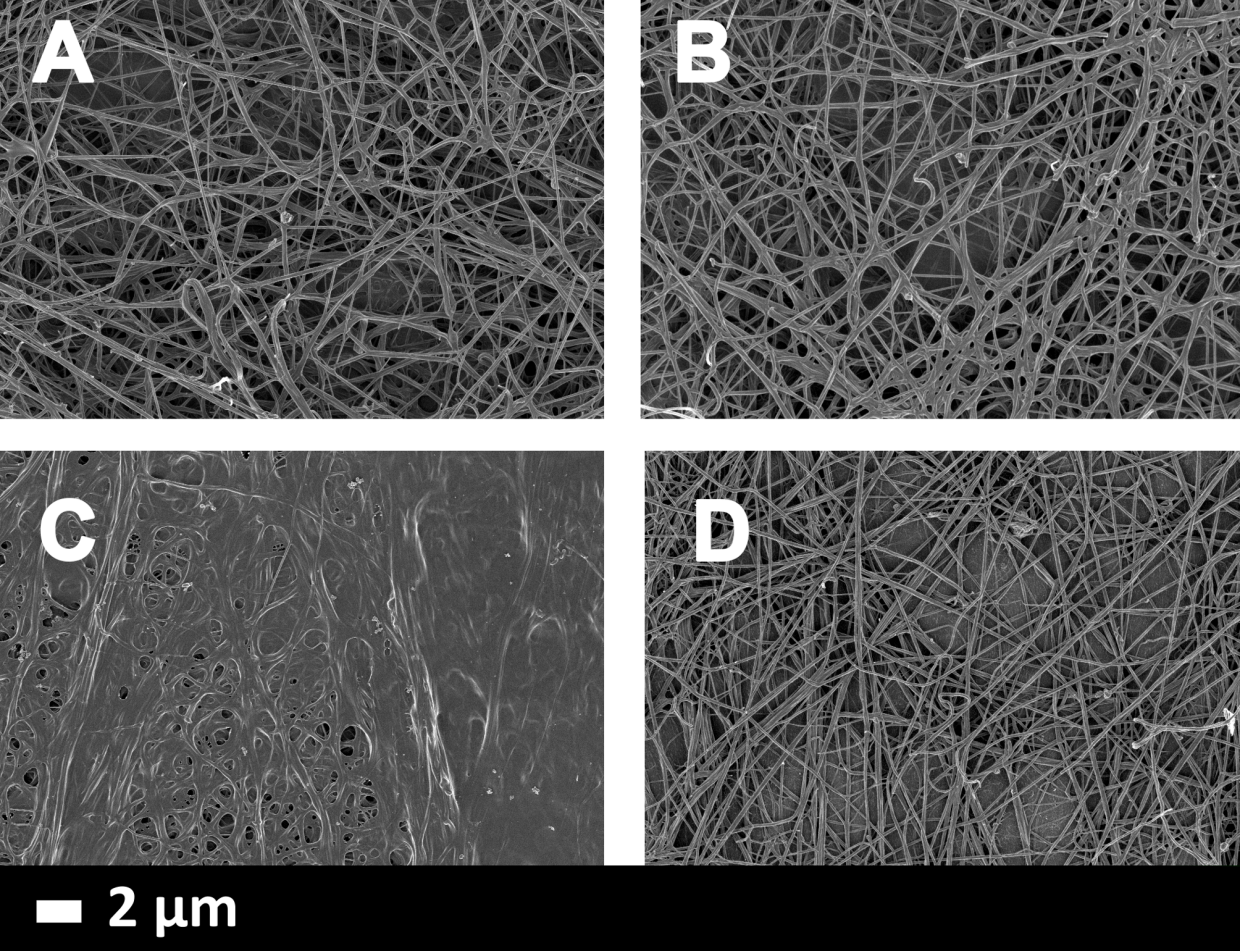
**A and B)** Clot structure from two healthy individuals, imaged in an SEM and showing a ‘normal’ ‘spaghetti’ or ‘noodle’ type of structure. All clots were created by adding thrombin to PPP. **C)** SEM micrographs of PPP from a Parkinson’s disease individuals with added thrombin, showing a ‘dense matted deposit’; **D)** PPP from same individual, but exposed to 2ng.L^-1^ LPS-binding protein followed by addition of thrombin; now showing a structure similar to that of healthy controls.

**Fig 2:**
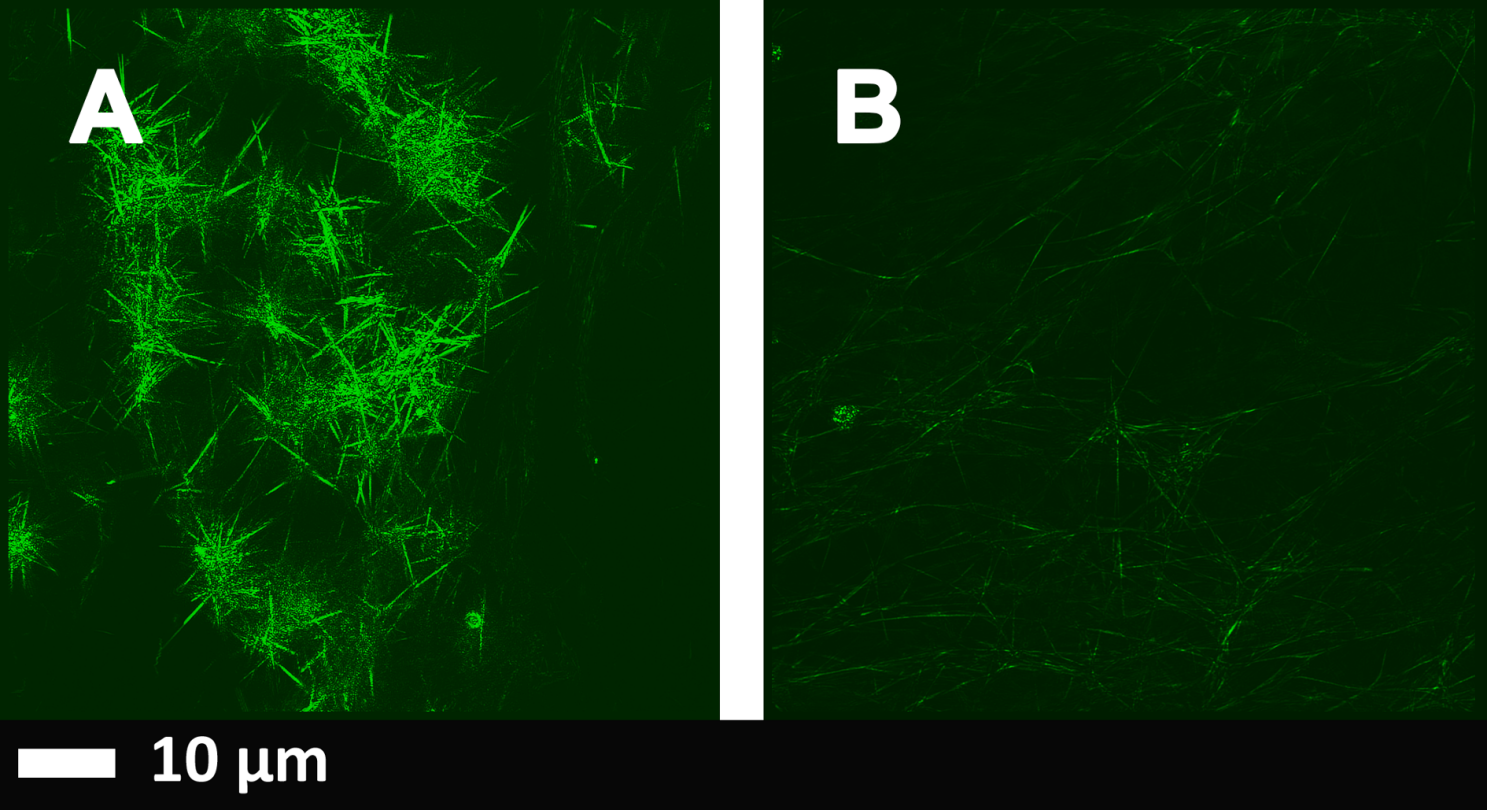
**A):** Airyscan micrographs of PPP with added thrombin to form extensive fibrin fibres from an individual with Parkinson’s disease (PD); **B)** PPP from the same individual, but exposed to 2ng.L^-1^ LPS-binding protein followed by addition of thrombin. Thioflavin T (ThT) (5 μM) was added before thrombin. Micrographs were taken with a Zeiss LSM 510 META confocal microscope with a Plan-Apochromat 63x/1.4 Oil DIC objective. ***LBP dramatically reduced the fluorescence seen in samples from patients with PD*.** Gain settings were kept the same during all data capturing and not changed for statistical analysis, but brightness and contrast was slightly adjusted for fig preparation.

**Fig 3:**
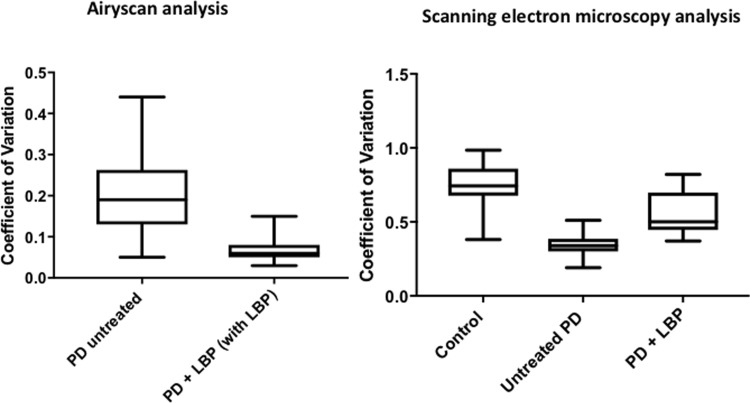
Boxplots of the distribution of the coefficients of variation in the pixel intensities of the SEM and Airyscan clot images.

**Fig 4:**
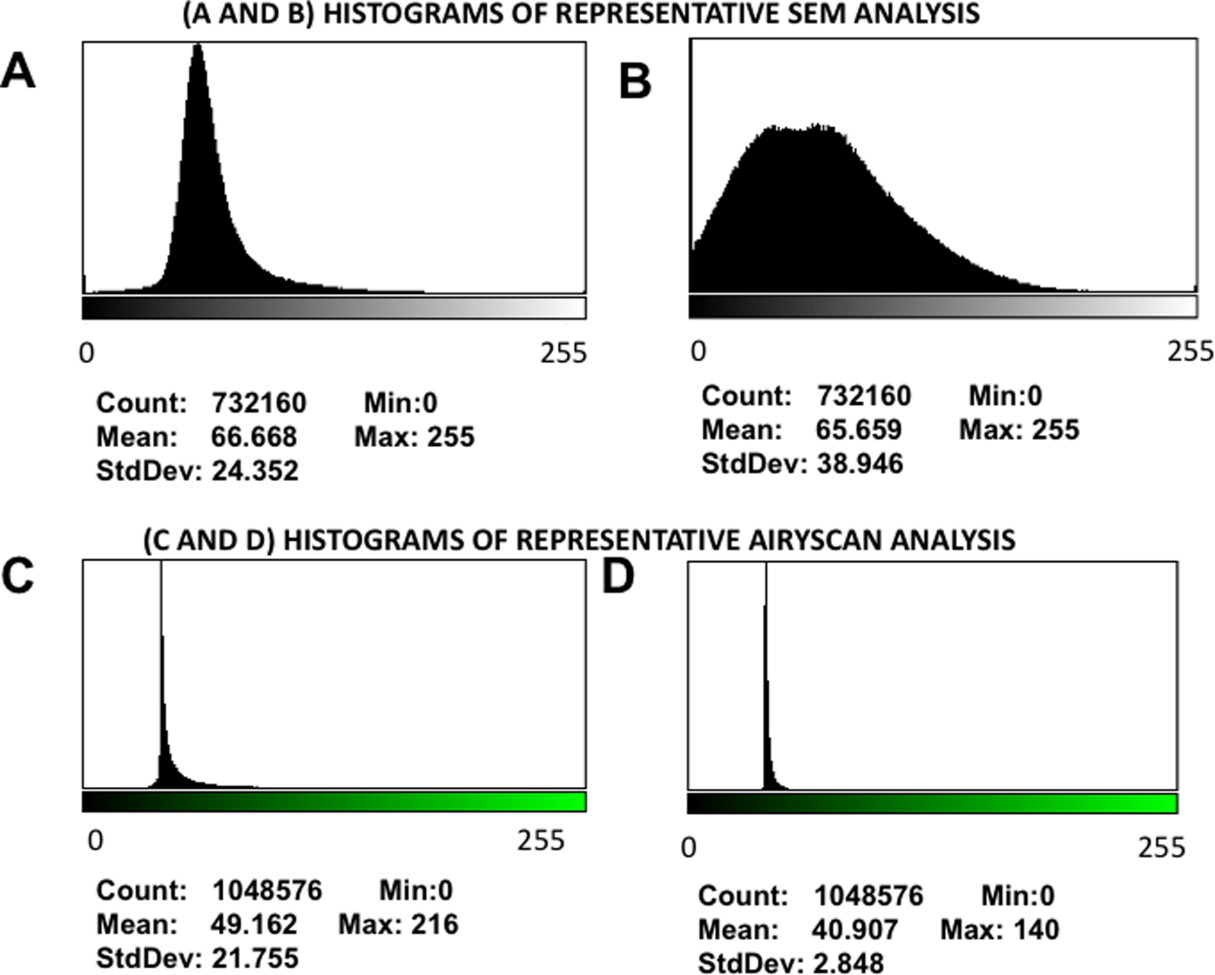
**A and B)** Representative histograms of the 8-bit intensity for a representative SEM clot from PPP of an individual with Parkinson’s disease and after addition of LBP, respectively. **C and D)** Representative histograms of the 8-bit intensity for a typical Airyscan clot from PPP of an individual with Parkinson’s disease and after addition of LBP, respectively.

Table 1 shows demographics for the healthy and the PD groups. Medication usage may influence the hematological system; however, previously we reviewed literature on PD medication interactions with the haematological system, and no significant interactions were previously reported (see [35] and Table 1). The median of the Hoehn and Yahr scale for the PD individuals were 2.5 (±0.43); suggesting that mostly, individuals participating in this study had mild bilateral PD symptoms at date of blood collection. Airyscan and SEM results are shown in Figs 1–4, for the healthy and PD individuals.

**Table 1:** Demographics and medication usage of Parkinson’s individuals.

HEALTHY INDIVIDUALS (N = 19)		
	**Gender**	**Age**
**Median; STD; %**	74% F; 26% M	All: 67.0 (±9.8) M: 67.0 (± 8.5) F: 67.0 (±10.5)
**PARKINSON’S DISEASE INDIVIDUALS (Hoehn and Yahr scale 2.5 (±0.43) (N = 26)**		
**Median; STD; %**	35% F: 65% M	All: 70 (±4.1) M: 70 (± 4.1) F: 71 (±4.2)
**PD MEDICATION USAGE**		
[Ropinirole (Requip®), SINEMET® (carbidopa-levodopa), Comtan® (entacapone), Pramipexole, Pexola®, Rasagiline, Azilect®,Stalevo® (carbidopa, levodopa, and entacapone), Madopar® (levodopa + benserazide), Carbilev® (carbidopa + levodopa) Amantadine].		

### Scanning electron microscopy of PPP clots

Fig 1A and 1B show how fibrin clots created from healthy PPP look like under a 10 000× machine magnification. Fibres have a spaghetti-like appearance, where individual fibres are visible. On the other hand, fibrin clots created from PPP of PD individuals all have a typical matted appearance, where individual fibres are entwined into a matted mass (see Fig 1C), indicative of hypercoagulation.

We recently suggested that this changed fibrin protein structure in inflammatory conditions like T2D, rheumatoid arthritis and others, are due to β-sheet-rich areas forming in the presence of both upregulated inflammatory markers, and particularly the presence or LPS [77]. We also showed that LPS added to healthy PPP created clots lead to hypercoagulation, and that the addition of LBP could reverse this pathological fibrin structure [77]. We additionally demonstrated that the pathological fibrin structure of T2D could be reversed with the addition of LBP (Pretorius *et al*., 2017a). Overall, we suggested that LBP protects the fibres from LPS damage by binding to LPS and, therefore, that LBP could decreaseβ-sheet-rich areas in T2D plasma.

Here we added LBP to PPP from PD individuals (see Fig 1D). We could show that in all our PD samples that a structural reversion to a state similar to clots created from healthy PPP could be obtained.

### Airyscan super-resolution microscopy of clots created from PPP

Previously we have noted that in healthy PPP, in the presence of ThT, little fluorescence was present, with only occasional very small patches of fluorescence [77, 78]. This might be because in all individuals (even if they are perceived to be healthy) there might be minor areas of misfolding of fibrin(ogen). We have also previously shown that when LPS had been incubated in healthy PPP, prior to the addition of thrombin, fluorescence was greatly enhanced, suggesting increased binding of ThT to β-sheet-rich areas on the fibrin(ogen) [77, 78]. From these results, we concluded that LPS binding causes the fibrinogen to polymerise into a form with a greatly increased amount of β-sheet (in the presence of thrombin), reflecting amyloid formation. This causes a strong fluorescence observable (when excited ca 440 nm) in the presence of ThT (see e.g. [70, 71]). In this paper, we also show β-sheet-rich areas in clots created from PPP of PD individuals (Fig 2).

Both Airyscan and SEM techniques are typically used only as qualitative methods. However, due to the increased fluorescence in clots prepared from PPP of PD, and also the more uniform and matted clot structure shown in SEM analysis of clots prepared from PD PPP, the variance between light and dark pixels are much less than seen in clots prepared from healthy PPP. We therefore propose using the coefficient of variation (CV) as our metric to quantify and discriminate between clots form healthy PPP and clots from PD PPP. We used ImageJ to calculate the mean and standard deviation of the intensity of the pixels in the images of the clot, using the histogram function, followed by the calculation of the coefficient of variation (i.e. SD/mean) of the intensity of the clot structure. Fig 3 shows boxplots of our results and Fig 4A–4D show examples of representative histograms of the 8-bit intensity for a typical SEM and confocal clot with and without LBP of a patient with PD (for the statistical analysis of results see Table 2).

**Table 2:** Data for Parkinson’s disease (PD) and healthy individuals showing P-values of coefficients of variation (CV) of the intensity of the pixels in the clot images using the one-way ANOVA test with Holm-Šídák Multiple Comparison’s Test comparing the mean of each column with the mean of every other column for the scanning electron microscopy analysis. The paired t-test was used for the Airyscan analysis (naïve PD vs. PD treated with LBP).

**SCANNING ELECTRON MICROSCOPY ANALYSIS (ANOVA)**					
	**Mean diff. of CVs**		**Adjusted P-value**		
Control (n = 19) vs PD (n = 26)	0.403		**<0.0001**		
Control (n = 19) vs PD + LBP (n = 26)	0.195		**<0.0001**		
PD (n = 26) vs PD + LBP (n = 26)	-0.21		**<0.0001**		
**AIRYSCAN ANALYSIS (PAIRED T-TEST)**					
	**Naïve PD**	**PD treated with LBP**		**P-value**	
**MEDIAN AND STD**	0.19 (± 0.09)	0.06 (± 0.03)		**<0.0001**	

Although LBP added to plasma from PD patients improved the structure of the fibrin(ogen) (see Fig 3, Airyscan boxplot), the PD with added LBP results are still statistically different from the control donors. LBP therefore does not allow the fibrin(ogen) to resemble fully that found in healthy individuals. We previously found in type 2 diabetes (T2D) [104] that when LBP is added to plasma from T2D, it did resemble that of healthy plasma.

We did not, in this paper, repeat Airyscan analysis with clots created with healthy PPP. However, we previously reported that in healthy individuals, little amyloid fluorescence is present when viewing clots with the added fluorescent marker, ThT [104–106].

## Discussion

Although we have observed anomalies in the kinds of fibrin fibres produced in the plasma of patients with various inflammatory diseases (e.g. [46, 49, 59, 75, 76, 78, 107–109]), this is the first time that we have observed fibrin amyloid in Parkinson’s Disease, as assessed by ThT staining, and its sensitivity (and that of fibres observed in the SEM) to LBP. While fibrin and α-synuclein can coaggregate [110], it is especially notable that the thrombin-dependence and SEM fibre sizes imply that the fibres we observe are essentially all made of fibrin. Although α-synuclein fibre formation in the substantia nigra is characteristic of PD, it can also occur extracellularly, and its removal may be of therapeutic benefit [111, 112]. The production may be driven by intestinal LPS [113], while gut microbiota-derived short-chain fatty acids may also have a role [11]. Because the addition of LBP to plasma of PD individuals improves the pathological state of the fibrin(ogen), we take it as indirect evidence that LPS has a role in this disease. So far as is known, the only action of LBP, and certainly at these concentrations, is to remove tiny amounts of LPS and related molecules, and only LPS has been shown (by us) to have this massive substoichiometric effect in affecting clotting [77, 104, 105]. Thus, targeting intestinal LPS possibly to not only reduce the production of extracellular α-synuclein, but also to target pathological clotting in PD, is therefore potentially a sensible treatment strategy to investigate further. The discovery that the addition of LBP to plasma influences the structure of fibrin(ogen), furthermore lends credence to the possible role of LPS in the aetiology of PD.

In terms of treatment [114, 115], we have previously discussed the potential role of iron chelators, both to stop the Fenton reaction [25, 26] and to inhibit microbial proliferation (e.g. [84–86, 91]), and iron chelators can definitely also inhibit the formation of dense matted (fibrin(ogen)) deposits (which is a hallmark of coagulopathies) (e.g. [27, 49, 58, 76, 116, 117]). We have also previously reported that the red blood cells (RBCs) of PD individuals are eryptotic [35], mainly due to the presence of circulating cytokines and increased oxidative stress [118, 119]. The possible removal of amyloid by LBP may also affect other cells of the haematological system, e.g. RBCs. It is now clear that treatment options worth exploring also include anticoagulants; as yet, however, the evidence for any effect of heparin is still awaited, due to Randomised Control Trials not having been done [120].

## Concluding remarks

We show here that (1) LBP is a potential treatment for PD-associated coagulopathies and (2) that the normalizing effect of LBP on fibrin(ogen) structure, strongly (albeit indirectly) points to the possibly important role of LPS in the aetiology of PD. Overall, the remarkable reversal of amyloid fibrin formation by LBP addition to the plasma of Parkinson’s Disease patients firstly present a novel strategy for treating PD-associated- coagulopathies and secondly implies strongly that LPS is naturally pre-existing in said plasma. Although almost all the LPS is bound to plasma proteins under normal conditions [86], including presumably to fibrinogen but in concentrations that are consequently hard to determine [86], it is known from experiments where LPS was added exogenously, that LBP molecules can inhibit the LPS-induced formation of the amyloid form of fibrin when thrombin is present [77]. Any endotoxin content of the PPP, ThT, LBP and thrombin is not known; however, the fact that the effect was fully reversed by LBP shows that any such endotoxin content must be negligible and/or irrelevant. So far as is known, the only action of LBP, and certainly at these concentrations, is to remove tiny amounts of LPS and related molecules, and only LPS has been shown (by us) to have this massive substoichiometric effect in affecting clotting. Consequently, the present work lends support to the idea (and evidence [121, 122]) that a dormant blood and tissue microbiome, capable of shedding LPS, is at least part of the aetiology of Parkinson’s Disease.
